# Supplementary material for: Intracellular Ca2+-Mediated AE2 Is Involved in the Vectorial Movement of HaCaT Keratinocyte
Source: Int J Mol Sci. 2020 Nov 10;21(22):8429. doi: 10.3390/ijms21228429 (PMC7698169; doi:10.3390/ijms21228429)
Supplement: Supplementary file 1 [file ijms-21-08429-s001.pdf]

Article

Intracellular Ca^{2+}-mediated AE2 is involved in the vectorial movement of HaCaT keratinocyte

Soyoung Hwang [1], Dong Min Shin [2,*] and Jeong Hee Hong [1,3*]

1 Department of Physiology, College of Medicine, Gachon University
2 Department of Oral Biology, Yonsei University College of Dentistry, Seoul 03722, Republic of Korea
3 Department of Health Sciences and Technology, GAIHST, Gachon University, 155 Getbeolro, Yeonsu-gu, Incheon 21999, Republic of Korea;

*Correspondence: minicleo@gachon.ac.kr (JHH); dmshin@yuhs.ac (DMS); Tel.:+82-32-899-6682 (JHH); Tel.:+82-2-2228-3051 (DMS); Fax:+82-32-899-6039 (JHH), Fax:+82-2-364-1085 (DMS)

Supplementary Figure S1. Hwang et al.

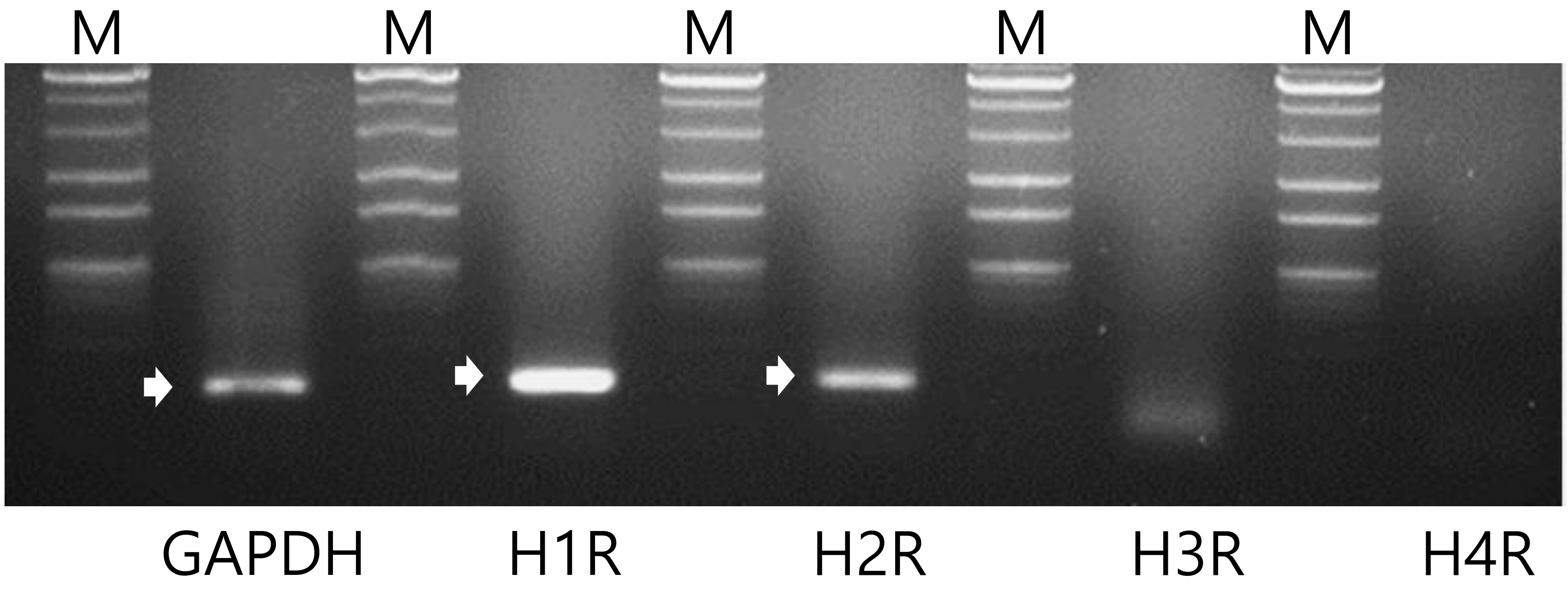

Supplementary Figure S1. The HaCaT keratinocytes were expressed Histamine family receptors.

The mRNA expression with Histamine family receptors (H1R, H2R, H3R and H4R, white arrows) in HaCaT cells. M: DNA ladder.
